# Association of social determinants of health with frailty, cognitive impairment, and self-rated health among older adults

**DOI:** 10.1371/journal.pone.0277290

**Published:** 2022-11-11

**Authors:** Vanessa Tan, Cynthia Chen, Reshma Aziz Merchant

**Affiliations:** 1 Saw Swee Hock School of Public Health, National University of Singapore, Singapore, Singapore; 2 Schaeffer Center for Health Policy and Economics, University of Southern California, Los Angeles, California, United States of America; 3 Department of Non-Communicable Disease Epidemiology, The London School of Hygiene & Tropical Medicine, London, United Kingdom; 4 Division of Geriatric Medicine, Department of Medicine, National University Hospital, Singapore, Singapore, Singapore; 5 Department of Medicine, Yong Loo Lin School of Medicine, National University of Singapore, Singapore, Singapore; Indian Institute of Technology Jodhpur, INDIA

## Abstract

**Background and objectives:**

Recently, the role of social determinants of health on frailty and dementia has received increased attention. The aim of the present study is to explore the association of social determinants on cognitive impairment, frailty, and self-rated health. As health is influenced by many factors, we also examine other health determinants including lifestyle, health seeking behaviour, socio-demographics, and multimorbidity in the analysis.

**Research design and methods:**

Cross-sectional analysis of the Healthy Older People Everyday (HOPE) study in Singapore was carried out on 998 older adults above the age of 65. We used forward stepwise multivariable logistic and linear regression analyses to assess the association of five health determinants (social determinants, lifestyle, health seeking behaviour, socio-demographics and multimorbidity) on frailty, cognitive impairment, and self-rated health.

**Results:**

Mean age of participants was 71.1 ± 0.2 years; 154 (15.4%) were cognitively impaired; 430 (43.1%) were pre-frail or frail; mean self-rated health was 80.4 ± 15.6. Social determinants contributed between 29% to 57% of the overall variation found in the full model with all five health determinants adjusted for. Participants with higher education had significantly lower odds of cognitive impairment and frailty. Leisure physical activity was significantly associated with lower odds of frailty and cognitive impairment, and better self-rated health.

**Discussion and implications:**

Understanding the dynamics of different health determinants is crucial to protect the vulnerable in an ageing population. Our study highlights the need for a multidimensional, multidisciplinary and multisectoral approach in the prevention of frailty, cognitive impairment, and associated disability.

## Introduction

Despite having improvements in healthcare and disease prevention, health disparities continue to persist. Old-age income poverty is closely related to population ageing as older adults are susceptible to economic insecurity due to retirement or declining capability in prolonging employment [1]. A recent report revealed that women and those above the age of 75 were found to be at greater risk of old-age income poverty [2]. As poverty impedes access to healthcare services, it increases the risk of adverse health outcomes.

To reduce health inequalities, the World Health Organization (WHO) Commission on Social Determinants of Health [3] was established to understand and address the underlying social factors affecting poor health. It refers to the circumstances in which people live in and include factors such as education, income, and housing, where better social circumstances are associated with better health [4]. As COVID-19 has magnified existing health disparities and their underlying social factors, social determinants have been increasingly regarded as an important health determinant.

Several studies have attempted to examine the relative contributions of health determinants. In the United States County Health Rankings model, 40% of health outcomes could be explained by social factors, 30% by health behaviours, 20% by medical care, and 10% by the physical environment [5]. Apart from social factors, health is also influenced by factors such as the environment, genetics, behaviour and quality of medical care [6]. Many of these health determinants are intricately intertwined. Education is believed to empower people to make healthier lifestyle choices and enhance employment opportunities, putting the individual in a better position to afford healthcare services [7]. Individuals with favourable social circumstances were found to be more likely to engage in preventive health seeking behaviour or participate in healthy behaviours, possibly due to increased awareness of health literacy or ability to afford healthcare services [8].

Ageing is a risk factor for chronic diseases, cognitive impairment, frailty, and associated disability [9]. Frailty is a state of decreased physiological reserve and heightened vulnerability to adverse events. Cognitive impairment and frailty are the main drivers of disability, and better predictor of adverse outcomes than age. Both are reversible if identified early before the onset of disability [10–12]. Many lifestyle factors and socio-economic factors have been linked to frailty and dementia such as education level, occupation, income, and wealth [13–16]. These factors are also linked to self-rated health, a measure of subjective health [17]. Understanding these dynamics may be crucial to protect the vulnerable in a rapidly ageing population and implement necessary intervention in the vulnerable group to delay the onset of frailty and cognitive impairment [11, 18].

Singapore is one of the most rapidly ageing societies in the world. Having transitioned from an ageing society to an aged society in 2019, Singapore is expected to become a super-aged society with 21% of its population above 65 by 2030 [19]. Older adults in Singapore mainly depend on intergenerational support for income security [20]. Public support schemes are available for those who lack family support or are unable to work but are limited, subjected to means-testing and may incur help-seeking costs [21]. Out of economic necessity, retirement may not be an option for some. For those still in the workforce, many of them are employed in lower paying jobs, with income insufficient to meet financial needs in Singapore [22, 23]. There are no official data on poverty in Singapore, but estimates on relative poverty rate, based on OECD’s definition, was found to be as high as 25% [24]. A GINI Coefficient of 0.452 also suggests that income disparity is prevalent in Singapore [25].

**Fig 1:**
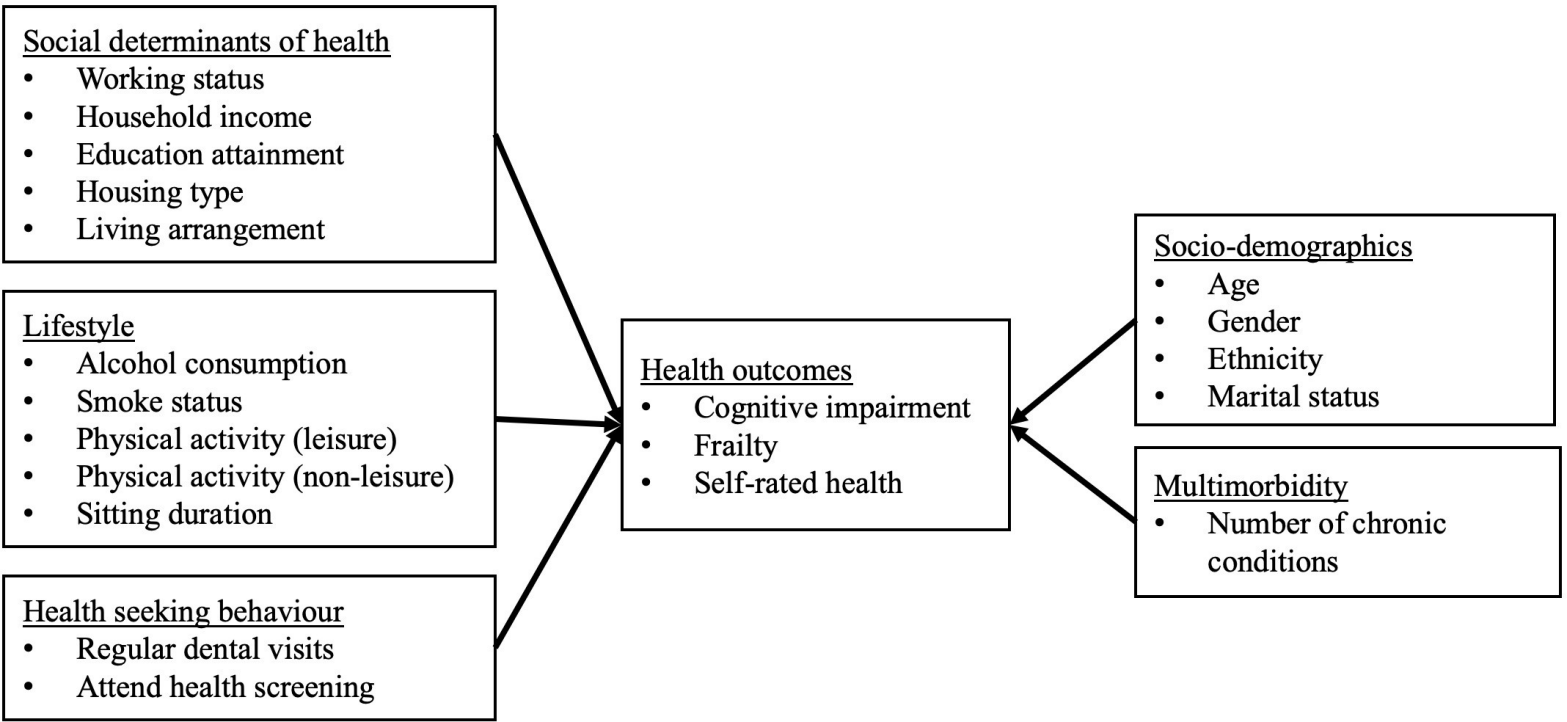
Conceptual framework.

The influence of social determinants of health among older adults has been widely documented, but it is unclear how much it accounts for health outcomes in a rapidly ageing Asian society like Singapore. Recently, the role of social determinants of health on frailty and dementia has received increased attention. The aim of the present study is to examine the association of social of determinants health on cognitive impairment, frailty, and self-rated health. As health outcomes are influenced by many factors, we also examine other health determinants including lifestyle, health seeking behaviour, socio-demographics, and multimorbidity in the analysis (**Fig 1**).

## Methods

### Study population

The Healthy Older People Everyday (HOPE) study, a sub cohort of the Singapore Population Health Studies–Community Health Study, is an epidemiological community-based study of older adults above the age of 65 from a defined geographical area in the Northwest region of Singapore. Trained interviewers conducted in-person interviews between April 2015 to August 2016 using a combination of interviewer administered questionnaires including the Mini Mental State Examination (MMSE) [26], EuroQol-visual analogue scale (EQ-VAS) [27], and five-item FRAIL scale [28]. Of the 1051 recruited older, 6 participants did not complete the questionnaire and 47 participants had missing data on demographics and medical history. They were excluded from the analysis. Of the remaining 998 participants, 374 participants had missing monthly household income. More information on recruitment, questionnaires and assessments are elaborated in prior publications [29]. All participants signed an informed consent form prior to participation in the study. This study was approved by the National Healthcare Group Institutional Review Board.

### Measures

#### (i) Social determinants of health

Social determinants of health are characterized by five variables: working status (yes, no), education attainment (primary and below vs. secondary and above), monthly household income (low: <S$2,000, mid: S$2,000–3,999, high: ≥S$4,000), housing type (3 room flat or smaller, 4 room flat, 5 room flat or bigger), and number of people living in the same household. Missing income data were imputed based on mean household monthly income by housing type.

#### (ii) Lifestyle

Lifestyle factors were assessed with questions pertaining to smoking status (never, ever, current smoker), alcohol consumption (no, once a month or less, more than once a month, participation in leisure and non-leisure physical activity (yes, no), and duration of sitting time (quartiles). Leisure physical activities refer to recreational activities including sports, exercise or walking during leisure time. Non-leisure physical activities refer to activities at work or household chores that require physical effort. Duration of sitting time was categorized into four quartiles (Quartile 1: ≤3 hours/day, Quartile 2: 3–4 hours/day, Quartile 3: 4–7 hours/day, Quartile 4: ≥7 hours/day).

#### (iii) Health seeking behaviour

Health seeking behaviour includes regular dental appointment (yes, no) and uptake in any recommended screening programs (yes, no). Regular dental appointment was defined as at least one oral health check-up within the past year. Participation in recommended screening programs refers to uptake in any of the following health screenings within the recommended period: blood stool test, colonoscopy, and mammogram. Health screening programs, allowing for early detection and diagnosis, are essential to promote independence and reduce the burden of disease on health systems. According to Ministry of Health in Singapore [30], clinical practice guidelines for blood stool test is recommended every three years, for colonoscopy every ten years, and for mammograph every two years.

#### (iv) Socio-demographic

Sociodemographic factors in this study included age (<75, ≥75 years old), gender (male, female), ethnicity (Chinese, Malay/Indian/Others), and marital status (married, single/separated/divorced/widowed).

#### (v) Multimorbidity

Self-reported chronic conditions included heart disease, diabetes, hyperlipidaemia, hypertension, stroke, and cancer. Multimorbidity was defined as the number of self-reported health conditions (no condition, 1 condition and ≥ 2 conditions).

### Outcomes

Cognitive functioning was assessed using the MMSE and cognitive impairment was defined using a cut-off of <24 [31]. Frailty was screened using the five-item FRAIL scale (Fatigue, Resistance, Ambulation, Illness, and Loss of Weight). A score of 0 represents robust, 1–2 represent pre-frail and 3–5 represent frail [28]. Frailty is defined as being pre-frail or frail in this study. Self-rated health was assessed using EQ-VAS where participants rate their overall health from a scale of 0 (poorest state of health) to 100 (best state of health) by looking at a 20 cm long scale.

### Statistical analysis

Data were summarized as means and standard deviation for continuous variables and as n (%) for categorical data. Chi-square test was performed to test the associations between categorical variables by monthly household income groups and ANOVA was used for continuous variables. Multivariable logistic regression was used to examine the association of monthly household income on cognitive impairment and frailty. Multivariable linear regression was used on self-rated health. Forward stepwise multivariable regressions of 17 variables from five health determinants, namely: (i) the social determinants of health, (ii) lifestyle, (iii) health seeking behaviour, (iv) socio-demographic factors, and (v) multimorbidity were conducted.

Each health outcomes were modelled through the specifications of five separate models. The first model included social determinants of health variables (working status, housing, household income, level of education attainment, number of people living with). The second model adjusted for additional lifestyle variables (smoking status, alcohol consumption, physical activity from work, leisure physical activity, sitting duration). The third model added health seeking behaviour (regular dental visits, regular screening tests). The fourth model added socio-demographic variables (age, gender, ethnicity, marital status). The fifth model added multimorbidity.

To assess how much variation was explained by the five health determinants, pseudo R^2^ statistics for logistic regression and adjusted R^2^ statistics for linear regression were reported for each model. To study how much each determinant of health contributed to the overall variability, the ratio of explained R^2^ was first calculated by dividing the R^2^ of the model by the fully adjusted model. Next, the change in ratio of explained R^2^ was computed by subtracting the ratio of explained R^2^ of the current model from the previous model. Statistical significance threshold was set at 5%. All statistical analyses were performed using Stata 15.1 [32].

## Results

**Fig 2:**
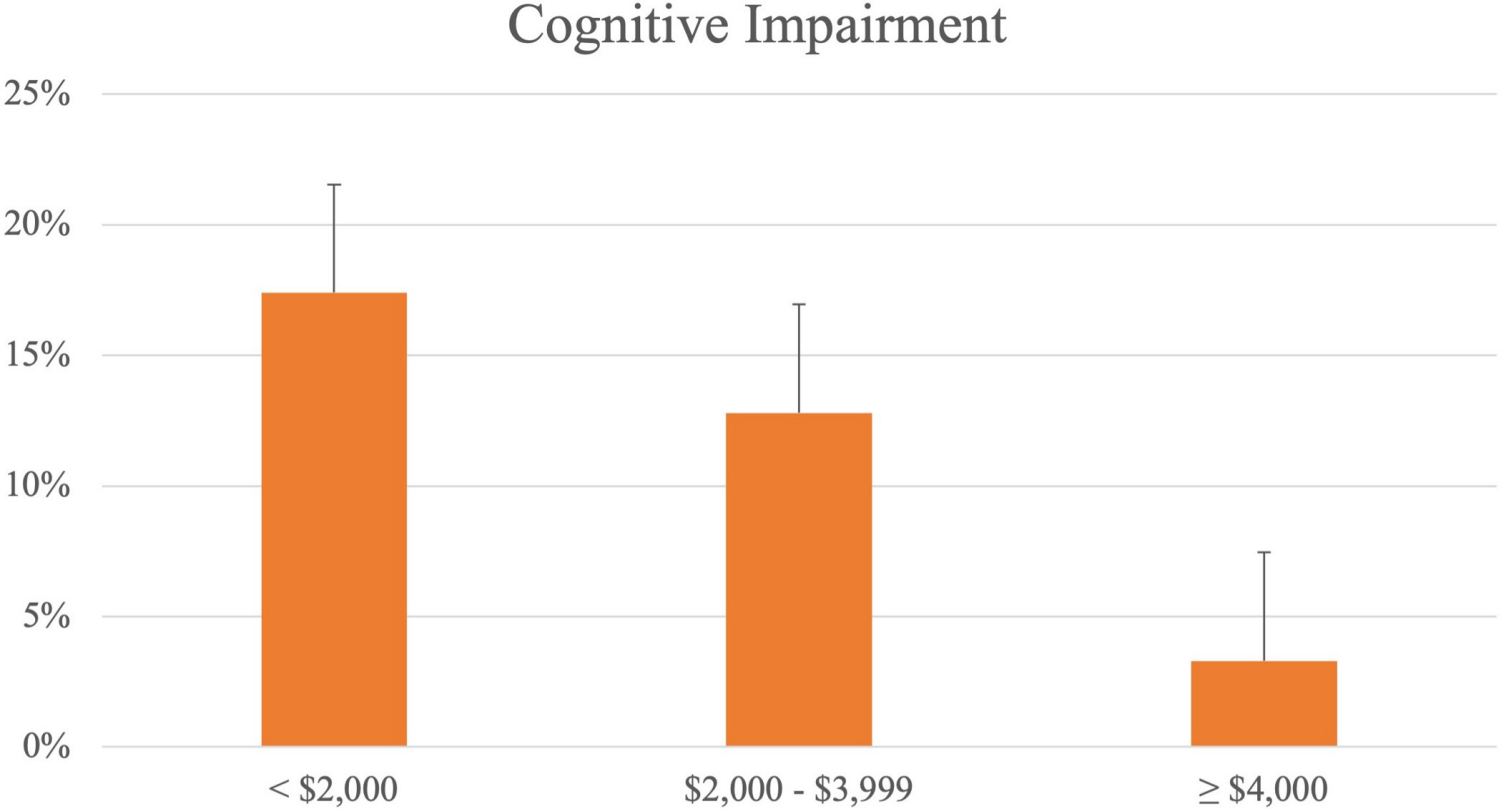
Percentage of participants with cognitive impairment, by income groups.

**Fig 3:**
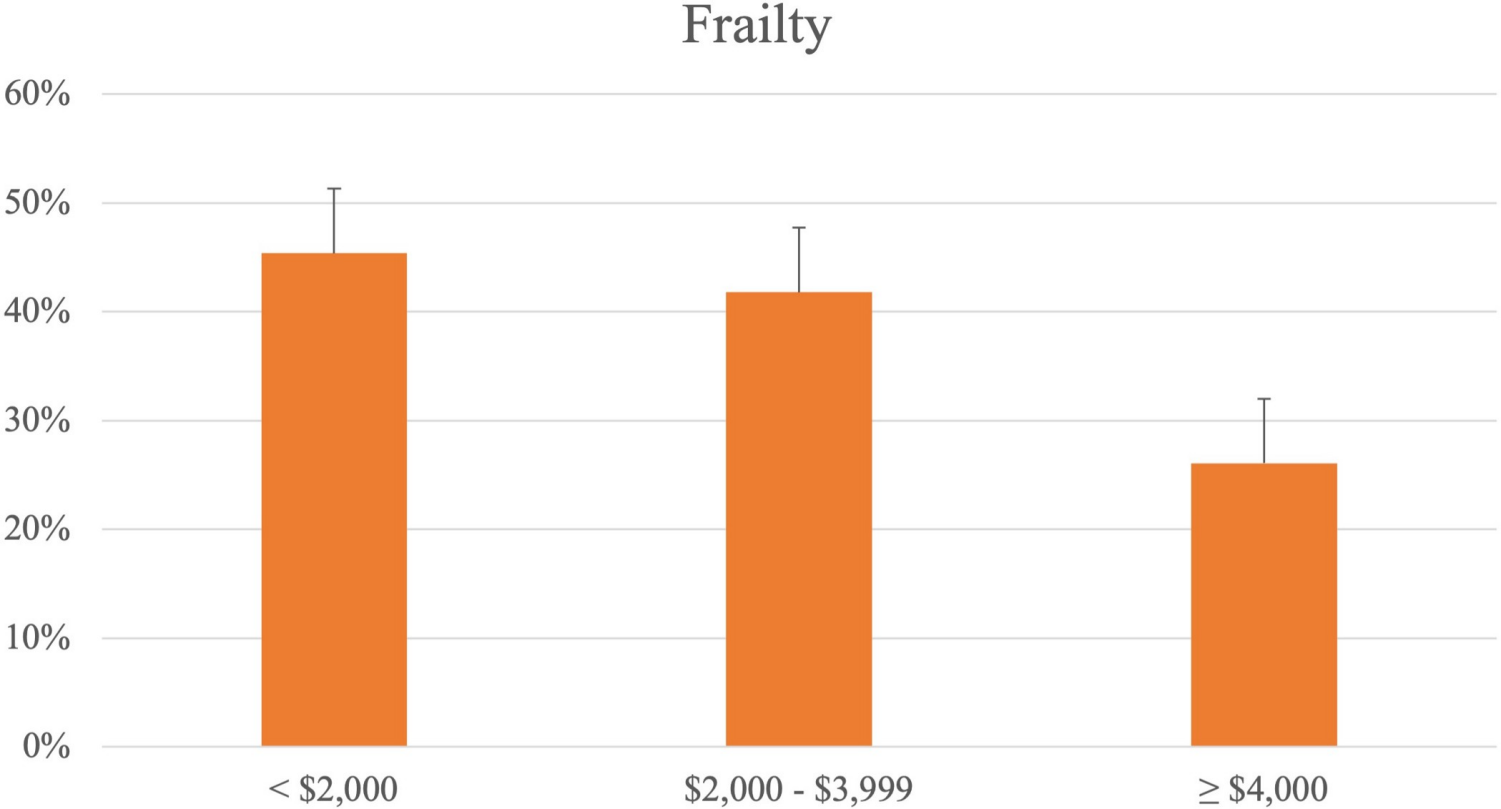
Percentage of participants with frailty, by income groups.

Across income groups, the proportion of participants with cognitive impairment was found to be highest in the low income (17.4%), followed by the middle income (12.8%) and the high income (3.30%) (**Fig 2**). The proportion of participants who were prefrail or frail also followed the same trend (low: 45.4%, mid: 41.8%, high: 26.1%) (**Fig 3**).

Participants with low income were more likely to have lower mean self-rated health (low 80.0 ± 16.0 vs. high 81.9 ± 13.8; p = 0.410) (**Table 1**). Compared to the high-income group, the low income was more likely to be older (low: 71.6 ± 5.52 vs. high: 69.1 ± 4.80; p < 0.001); more likely to be female (low 60.7% vs. high 39.1%; p < 0.001); more likely to be single/separated/divorced/widowed (low 31.5% vs. high 20.7%; p = 0.012); more likely to live with lesser people (low 2.49 ± 1.89 vs. high 3.04 ± 1.77; p = 0.017); more likely to have attained primary level of education or below (low 67.3% vs. high 35.9%; p < 0.001); less likely to be working (low 24.4% vs. high 52.2%; p < 0.001); less likely to consume alcohol (low 75.3% vs. high 59.8%; p = 0.004); less likely to engage in leisure physical activity (low 23.1% vs. high 33.7%; p = 0.050); less likely to engage in non-leisure physical activity (low 26.2% vs. high 34.8%; p = 0.250); less likely to have regular dental visits (low 63.0% vs. high 46.7%; p = 0.010; and less likely to attend health screening (low 56.3% vs. high 54.3%; p = 0.200) (**Table 1**).

**Table 1:** Characteristics by monthly household income.

		Total	< $2,000	$2,000 - $3,999	≥$4,000	p-value
		N = 998	N = 765	N = 141	N = 92	
Age						<0.001
	< 75 years old	762 (76.4%)	558 (72.9%)	121 (85.8%)	83 (90.2%)	
	≥ 75 years old	236 (23.6%)	207 (27.1%)	20 (14.2%)	9 (9.8%)	
Gender						<0.001
	Male	430 (43.1%)	301 (39.3%)	73 (51.8%)	56 (60.9%)	
	Female	568 (56.9%)	464 (60.7%)	68 (48.2%)	36 (39.1%)	
Ethnicity						<0.001
	Chinese	798 (80.0%)	626 (81.8%)	94 (66.7%)	78 (84.8%)	
	Malay/Indian/Others	200 (20.0%)	139 (18.2%)	47 (33.3%)	14 (15.2%)	
Marital status						0.012
	Married	707 (70.8%)	524 (68.5%)	110 (78.0%)	73 (79.3%)	
	Single/separated/divorced/widowed	291 (29.2%)	241 (31.5%)	31 (22.0%)	19 (20.7%)	
Number of people living with		2.56 (1.82)	2.49 (1.89)	2.66 (1.35)	3.04 (1.77)	0.017
Work status						<0.001
	Working	307 (30.8%)	187 (24.4%)	72 (51.1%)	48 (52.2%)	
	Not working	691 (69.2%)	578 (75.6%)	69 (48.9%)	44 (47.8%)	
Education level						<0.001
	Primary and below	617 (61.8%)	515 (67.3%)	69 (48.9%)	33 (35.9%)	
	Secondary and above	381 (38.2%)	250 (32.7%)	72 (51.1%)	59 (64.1%)	
Alcohol consumption (Past year)						0.004
	No	730 (73.1%)	576 (75.3%)	99 (70.2%)	55 (59.8%)	
	Once a month or less	165 (16.5%)	118 (15.4%)	21 (14.9%)	26 (28.3%)	
	More than once a month	103 (10.3%)	71 (9.3%)	21 (14.9%)	11 (12.0%)	
Smoke status						0.013
	Never smoker	714 (71.5%)	554 (72.4%)	89 (63.1%)	71 (77.2%)	
	Ever smoker	194 (19.4%)	137 (17.9%)	42 (29.8%)	15 (16.3%)	
	Current smoker	90 (9.0%)	74 (9.7%)	10 (7.1%)	6 (6.5%)	
Physical activity (Leisure)						0.050
	No	750 (75.2%)	588 (76.9%)	101 (71.6%)	61 (66.3%)	
	Yes	248 (24.8%)	177 (23.1%)	40 (28.4%)	31 (33.7%)	
Physical activity (Non-leisure)						0.250
	No	724 (72.5%)	560 (73.2%)	104 (73.8%)	60 (65.2%)	
	Yes	274 (27.5%)	205 (26.8%)	37 (26.2%)	32 (34.8%)	
Sitting duration (Quartile)						0.098
	Q1	352 (35.3%)	280 (36.6%)	49 (34.8%)	23 (25.0%)	
	Q2	157 (15.7%)	120 (15.7%)	27 (19.1%)	10 (10.9%)	
	Q3	278 (27.9%)	210 (27.5%)	36 (25.5%)	32 (34.8%)	
	Q4	211 (21.1%)	155 (20.3%)	29 (20.6%)	27 (29.3%)	
Regular dental visits						0.010
	No	612 (61.3%)	482 (63.0%)	87 (61.7%)	43 (46.7%)	
	Yes	386 (38.7%)	283 (37.0%)	54 (38.3%)	49 (53.3%)	
Attend health screening						0.200
	No	549 (55.0%)	431 (56.3%)	68 (48.2%)	50 (54.3%)	
	Yes	449 (45.0%)	334 (43.7%)	73 (51.8%)	42 (45.7%)	
Presence of chronic conditions						0.380
	No condition	209 (20.9%)	158 (20.7%)	33 (23.4%)	18 (19.6%)	
	1 condition	268 (26.9%)	203 (26.5%)	33 (23.4%)	32 (34.8%)	
	≥ 2 conditions	521 (52.2%)	404 (52.8%)	75 (53.2%)	42 (45.7%)	
Cognitive impairment (MMSE)						0.001
	No (≥ 24)	844 (84.6%)	632 (82.6%)	123 (87.2%)	89 (96.7%)	
	Yes (< 24)	154 (15.4%)	133 (17.4%)	18 (12.8%)	3 (3.3%)	
Frail status						0.002
	Robust	568 (56.9%)	418 (54.6%)	82 (58.2%)	68 (73.9%)	
	Prefrail or frail	430 (43.1%)	347 (45.4%)	59 (41.8%)	24 (26.1%)	
Self-rated health (EQ-VAS)		80.4 (15.6)	80.0 (16.0)	81.3 (14.5)	81.9 (13.8)	0.410

*Notes*: MMSE = Mini Mental State Examination; EQ-VAS = EuroQol-visual analogue scale.

Chi-square and t-test statistics were used to evaluate significant differences in means and proportions.

### Cognitive impairment

**Table 2** presents multivariable analyses to examine the association of the five health determinants with cognitive impairment. Social determinants contributed to the highest change in the explained variation in cognitive impairment. Out of the total variance observed in the full model (Model 5), social determinant factors accounted for 57% of the explained variation found in cognitive impairment.

**Table 2:** Odds ratio (95% confidence interval) on cognitive impairment.

		Model 1 (Social Determinants of Health)	Model 2 (Model 1 + Lifestyle)	Model 3 (Model 2 + Health Seeking Behaviour)	Model 4 (Model 3 + Socio-demographics)	Model 5 (Model 4 + Multimorbidity)
		OR (95% CI)	OR (95% CI)	OR (95% CI)	OR (95% CI)	OR (95% CI)
Working status						
	Working	1 (ref)	1 (ref)	1 (ref)	1 (ref)	1 (ref)
	Not working	2.99*** (1.80, 4.98)	2.73*** (1.62, 4.61)	2.75*** (1.62, 4.66)	2.06** (1.19, 3.55)	2.06** (1.19, 3.55)
Household income						
	≥$4,000	1 (ref)	1 (ref)	1 (ref)	1 (ref)	1 (ref)
	$2,000 - $3,999	3.51 (0.97, 12.69)	3.5 (0.94, 13.00)	3.46 (0.94, 12.80)	2.66 (0.69, 10.19)	2.66 (0.69, 10.20)
	< $2,000	3.38* (1.00, 11.41)	3.49* (1.01, 12.01)	3.36 (0.98, 11.51)	3.04 (0.87, 10.63)	3.05 (0.87, 10.66)
Education attainment						
	Primary and below	1 (ref)	1 (ref)	1 (ref)	1 (ref)	1 (ref)
	Secondary and above	0.24*** (0.15, 0.41)	0.23*** (0.14, 0.40)	0.25*** (0.15, 0.44)	0.30*** (0.17, 0.53)	0.30*** (0.17, 0.53)
Housing						
	HDB 3 room flat or smaller	1 (ref)	1 (ref)	1 (ref)	1 (ref)	1 (ref)
	HDB 4 room flat	0.54** (0.34, 0.86)	0.62* (0.38, 1.00)	0.60* (0.37, 0.98)	0.75 (0.44, 1.25)	0.75 (0.45, 1.25)
	HDB 5 room flat or bigger	0.31*** (0.18, 0.55)	0.45** (0.25, 0.82)	0.46* (0.25, 0.84)	0.57 (0.30, 1.09)	0.57 (0.30, 1.09)
Number of people living with		1.21*** (1.09, 1.34)	1.18** (1.06, 1.31)	1.19** (1.06, 1.32)	1.17** (1.04, 1.31)	1.17** (1.04, 1.31)
Alcohol consumption						
	No		1 (ref)	1 (ref)	1 (ref)	1 (ref)
	Once a month or less		0.59 (0.31, 1.11)	0.65 (0.34, 1.24)	0.84 (0.43, 1.64)	0.83 (0.43, 1.62)
	More than once a month		0.76 (0.35, 1.62)	0.77 (0.36, 1.65)	1.09 (0.49, 2.41)	1.1 (0.50, 2.45)
Smoking status						
	Never smoker		1 (ref)	1 (ref)	1 (ref)	1 (ref)
	Ever smoker		0.73 (0.43, 1.23)	0.69 (0.41, 1.17)	1.09 (0.57, 2.09)	1.1 (0.57, 2.11)
	Current smoker		0.67 (0.32, 1.40)	0.6 (0.28, 1.27)	1.06 (0.45, 2.49)	1.06 (0.45, 2.49)
Physical activity (Leisure)						
	No		1 (ref)	1 (ref)	1 (ref)	1 (ref)
	Yes		0.54* (0.30, 1.00)	0.6 (0.33, 1.11)	0.63 (0.33, 1.18)	0.63 (0.33, 1.18)
Physical activity (Non-leisure)						
	No		1 (ref)	1 (ref)	1 (ref)	1 (ref)
	Yes		0.57* (0.34, 0.97)	0.58* (0.34, 0.99)	0.56* (0.32, 0.96)	0.56* (0.33, 0.96)
Sitting duration (Quartile)						
	Q1		1 (ref)	1 (ref)	1 (ref)	1 (ref)
	Q2		0.78 (0.43, 1.40)	0.77 (0.43, 1.39)	0.91 (0.50, 1.67)	0.92 (0.50, 1.68)
	Q3		0.91 (0.55, 1.52)	0.93 (0.56, 1.56)	0.88 (0.52, 1.50)	0.88 (0.52, 1.49)
	Q4		1.95** (1.19, 3.20)	1.96** (1.19, 3.23)	1.73* (1.03, 2.93)	1.74* (1.02, 2.94)
Regular dental visits						
	No			1 (ref)	1 (ref)	1 (ref)
	Yes			0.67 (0.44, 1.04)	0.68 (0.43, 1.06)	0.67 (0.43, 1.06)
Attend health screening						
	No			1 (ref)	1 (ref)	1 (ref)
	Yes			0.66* (0.44, 0.98)	0.68 (0.45, 1.03)	0.68 (0.45, 1.03)
Age						
	< 75 years old				1 (ref)	1 (ref)
	≥ 75 years old				2.03** (1.31, 3.13)	2.06** (1.32, 3.20)
Gender						
	Male				1 (ref)	1 (ref)
	Female				2.15* (1.16, 3.96)	2.15* (1.17, 3.98)
Ethnicity						
	Chinese				1 (ref)	1 (ref)
	Malay/Indian/Others				1.93** (1.19, 3.14)	1.96** (1.20, 3.19)
Marital status						
	Married				1 (ref)	1 (ref)
	Single/Separated/Divorced/Widowed				1.74* (1.13, 2.70)	1.75* (1.13, 2.71)
Multimorbidity						
	No condition					1 (ref)
	1 condition					1.15 (0.63, 2.10)
	≥ 2 conditions					1.03 (0.60, 1.77)
R^2^		13%	17%	18%	23%	23%
Ratio of explained R^2^		57%	74%	78%	100%	100%
Change in ratio of explained R^2^		57%	17%	4%	22%	0%

*Notes*: CI = confidence interval; OR = odds ratio.

*p < 0.05;

**p < 0.01;

***p < 0.001

Individuals who were less privileged was associated with higher odds of cognitive impairment. Compared to those with lower level of education attainment, those with higher education were less likely to be cognitively impaired. This significance remained in the fully adjusted model (Model 5: OR = 0.30, 95% CI 0.17–0.53) (**Table 2**). Similarly, compared to those residing in a smaller flat, those residing in bigger apartments had lower odds of cognitive impairment. In the fully adjusted model, the housing gradient remains, but was no longer statistically significant.

Within lifestyle factors, only non-leisure physical activity (Model 5: OR = 0.56, 95% CI 0.33–0.96) and long sitting duration (Model 5: OR = 1.74, 95% CI 1.02–2.94) was significantly associated with cognitive impairment in the full model. Significant associations were observed in all socio-demographic factors with cognitive impairment while no significant association was found for health seeking behaviour factors and multimorbidity in the full model.

### Frailty

**Table 3** presents multivariable analyses for frailty. Social determinants of health accounted for the highest change in total variance found in frailty from the full model. Social determinants of health contributed 43% of the variation found in the model for frailty. Among the variables of social determinants, working status, housing income and level of education attainment were found to be significantly associated with frailty. However, only level of education attainment and low household income remained significantly associated with frailty in the fully adjusted model. Older adults who had higher education were less likely to be prefrail or frail (Model 5: OR = 0.72, 95% CI 0.53–0.98). Compared to the higher household income group, older adults with lower monthly household income were more likely to be prefrail or frail (Model 5: OR = 1.77, 95% CI 1.01–3.09). Interestingly, engaging in leisure physical activity was found to be significantly associated with lower odds of frailty while engaging in non-leisure physical activity was significantly associated with higher odds of frailty (Model 5: OR = 0.66, 95% CI 0.47–0.93 for leisure physical activity; OR = 1.91, 05% CI 1.39–2.63 for non-leisure physical activity). Those who were older was associated with higher odds of frailty (Model 5: OR = 1.37, 95% CI 1.03–1.83). More than two chronic conditions were also associated with higher odds of frailty (Model 5: OR = 1.93, 95% CI 1.35–2.76).

**Table 3:** Odds ratio (95% confidence interval) on frailty.

		Model 1 (Social Determinants of Health)	Model 2 (Model 1 + Lifestyle)	Model 3 (Model 2 + Health Seeking Behaviour)	Model 4 (Model 3 + Socio- demographics)	Model 5 (Model 4 + Multimorbidity)
		OR (95% CI)	OR (95% CI)	OR (95% CI)	OR (95% CI)	OR (95% CI)
Working status						
	Working	1 (ref)	1 (ref)	1 (ref)	1 (ref)	1 (ref)
	Not working	1.50** (1.12, 2.01)	1.51** (1.12, 2.04)	1.50** (1.11, 2.03)	1.34 (0.98, 1.83)	1.31 (0.96, 1.79)
Household income						
	≥$4,000	1 (ref)	1 (ref)	1 (ref)	1 (ref)	1 (ref)
	$2,000 - $3,999	1.97* (1.10, 3.52)	2.02* (1.10, 3.68)	1.99* (1.09, 3.64)	1.74 (0.94, 3.21)	1.72 (0.93, 3.20)
	< $2,000	1.93* (1.13, 3.28)	1.98* (1.15, 3.42)	1.97* (1.14, 3.40)	1.78* (1.02, 3.11)	1.77* (1.01, 3.09)
Education attainment						
	Primary and below	1 (ref)	1 (ref)	1 (ref)	1 (ref)	1 (ref)
	Secondary and above	0.65** (0.49, 0.85)	0.68** (0.51, 0.90)	0.69* (0.51, 0.92)	0.73* (0.54, 0.99)	0.72* (0.53, 0.98)
Housing						
	HDB 3 room flat or smaller	1 (ref)	1 (ref)	1 (ref)	1 (ref)	1 (ref)
	HDB 4 room flat	0.67* (0.46, 0.96)	0.66* (0.46, 0.95)	0.66* (0.45, 0.95)	0.71 (0.49, 1.04)	0.72 (0.50, 1.06)
	HDB 5 room flat or bigger	0.74 (0.50, 1.10)	0.72 (0.48, 1.09)	0.72 (0.48, 1.08)	0.8 (0.52, 1.22)	0.82 (0.53, 1.26)
Number of people living with		1.21*** (1.09, 1.34)	1.07 (0.99, 1.16)	1.06 (0.98, 1.15)	1.06 (0.98, 1.15)	1.06 (0.97, 1.14)
Alcohol consumption						
	No		1 (ref)	1 (ref)	1 (ref)	1 (ref)
	Once a month or less		0.64* (0.44, 0.92)	0.64* (0.44, 0.93)	0.71 (0.48, 1.04)	0.72 (0.49, 1.07)
	More than once a month		0.96 (0.61, 1.51)	0.96 (0.61, 1.51)	1.07 (0.67, 1.71)	1.17 (0.73, 1.88)
Smoking status						
	Never smoker		1 (ref)	1 (ref)	1 (ref)	1 (ref)
	Ever smoker		1.12 (0.80, 1.58)	1.12 (0.80, 1.58)	1.23 (0.82, 1.84)	1.18 (0.78, 1.76)
	Current smoker		0.63 (0.39, 1.03)	0.63 (0.38, 1.03)	0.74 (0.43, 1.27)	0.74 (0.43, 1.28)
Physical activity (Leisure)						
	No		1 (ref)	1 (ref)	1 (ref)	1 (ref)
	Yes		0.65* (0.46, 0.90)	0.65* (0.46, 0.91)	0.67* (0.48, 0.94)	0.66* (0.47, 0.93)
Physical activity (Non-leisure)						
	No		1 (ref)	1 (ref)	1 (ref)	1 (ref)
	Yes		1.89*** (1.39, 2.59)	1.89*** (1.39, 2.59)	1.90*** (1.39, 2.61)	1.91*** (1.39, 2.63)
Sitting duration (Quartile)						
	Q1		1 (ref)	1 (ref)	1 (ref)	1 (ref)
	Q2		1.29 (0.87, 1.91)	1.29 (0.87, 1.92)	1.33 (0.90, 1.99)	1.34 (0.90, 2.00)
	Q3		1.26 (0.91, 1.76)	1.27 (0.91, 1.77)	1.24 (0.88, 1.74)	1.18 (0.84, 1.66)
	Q4		1.24 (0.86, 1.80)	1.24 (0.85, 1.79)	1.18 (0.81, 1.72)	1.07 (0.73, 1.57)
Regular dental visits						
	No			1 (ref)	1 (ref)	1 (ref)
	Yes			0.92 (0.70, 1.22)	0.93 (0.70, 1.23)	0.93 (0.70, 1.24)
Attend health screening						
	No			1 (ref)	1 (ref)	1 (ref)
	Yes			1.05 (0.80, 1.37)	1.1 (0.83, 1.45)	1.09 (0.83, 1.44)
Age						
	< 75 years old				1 (ref)	1 (ref)
	≥ 75 years old				1.45* (1.09, 1.92)	1.37* (1.03, 1.83)
Gender						
	Male				1 (ref)	1 (ref)
	Female				1.27 (0.88, 1.83)	1.26 (0.87, 1.83)
Ethnicity						
	Chinese				1 (ref)	1 (ref)
	Malay/Indian/Others				1.49* (1.04, 2.13)	1.41 (0.98, 2.03)
Marital status						
	Married				1 (ref)	1 (ref)
	Single/Separated/Divorced/Widowed				1.11 (0.81, 1.52)	1.11 (0.80, 1.53)
Multimorbidity						
	No condition					1 (ref)
	1 condition					1.18 (0.79, 1.76)
	≥ 2 conditions					1.93*** (1.35, 2.76)
R^2^		3%	5%	5%	6%	7%
Ratio of explained R^2^		43%	71%	71%	86%	100%
Change in ratio of explained R^2^		43%	29%	0%	14%	14%

*Notes*: CI = confidence interval; OR = odds ratio.

*p < 0.05;

**p < 0.01;

***p < 0.001

### Self-rated health

The multivariable analyses result examining the association between the five health determinants on self-rated health is shown in **Table 4**. Social determinants of health accounted for 29% in the full model. In the full model, working status, non-leisure physical activity, longer sitting duration, and having more than two chronic conditions were negatively significantly associated with self-rated health. In contrast, significant positive association were observed between leisure physical activity and Malay/Indian/Others ethnicity on self-rated health.

**Table 4:** Regression coefficients from multiple linear regression analysis on self-rated health.

		Model 1 (Social Determinants of Health)	Model 2 (Model 1 + Lifestyle)	Model 3 (Model 2 + Health Seeking Behaviour)	Model 4 (Model 3 + Socio-demographics)	Model 5 (Model 4 + Multimorbidity)
		β (95% CI)	β (95% CI)	β (95% CI)	β (95% CI)	β (95% CI)
Working status						
	Working	(ref)	(ref)	(ref)	(ref)	(ref)
	Not working	-3.88*** (-6.05, -1.70)	-4.23*** (-6.42, -2.03)	-4.24*** (-6.44, -2.04)	-4.06*** (-6.31, -1.81)	-3.79*** (-6.03, -1.55)
Household income						
	≥$4,000	(ref)	(ref)	(ref)	(ref)	(ref)
	$2,000 - $3,999	-0.47 (-4.57, 3.63)	-0.67 (-4.80, 3.45)	-0.82 (-4.95, 3.32)	-1.59 (-5.75, 2.56)	-1.29 (-5.42, 2.84)
	< $2,000	-1.76 (-5.45, 1.93)	-1.72 (-5.43, 1.98)	-1.79 (-5.49, 1.92)	-1.76 (-5.47, 1.95)	-1.48 (-5.17, 2.21)
Education attainment						
	Primary and below	(ref)	(ref)	(ref)	(ref)	(ref)
	Secondary and above	-0.84 (-2.93, 1.24)	-1.00 (-3.11, 1.12)	-1.17 (-3.32, 0.97)	-1.55 (-3.77, 0.66)	-1.48 (-3.69, 0.72)
Housing						
	HDB 3 room flat or smaller	(ref)	(ref)	(ref)	(ref)	(ref)
	HDB 4 room flat	-2.01 (-4.76, 0.74)	-2.15 (-4.90, 0.61)	-2.13 (-4.89, 0.63)	-1.42 (-4.20, 1.37)	-1.53 (-4.30, 1.24)
	HDB 5 room flat or bigger	-0.14 (-3.11, 2.84)	-0.26 (-3.32, 2.80)	-0.39 (-3.45, 2.67)	0.60 (-2.53, 3.74)	0.57 (-2.54, 3.69)
Number of people living with		0.39 (-0.18, 0.97)	0.55 (-0.03, 1.13)	0.55 (-0.03, 1.13)	0.41 (-0.17, 1.00)	0.46 (-0.13, 1.04)
Alcohol consumption						
	No		(ref)	(ref)	(ref)	(ref)
	Once a month or less		0.51 (-2.15, 3.17)	0.23 (-2.46, 2.92)	1.17 (-1.57, 3.92)	0.96 (-1.77, 3.69)
	More than once a month		1.15 (-2.18, 4.48)	1.11 (-2.22, 4.44)	2.02 (-1.35, 5.38)	1.74 (-1.63, 5.10)
Smoking status						
	Never smoker		(ref)	(ref)	(ref)	(ref)
	Ever smoker		-1.31 (-3.83, 1.21)	-1.14 (-3.67, 1.38)	-0.44 (-3.35, 2.46)	-0.10 (-2.99, 2.79)
	Current smoker		-1.18 (-4.72, 2.36)	-0.80 (-4.38, 2.77)	-0.10 (-3.98, 3.78)	-0.04 (-3.89, 3.82)
Physical activity (Leisure)						
	No		(ref)	(ref)	(ref)	(ref)
	Yes		2.2 (-0.20, 4.61)	1.99 (-0.43, 4.41)	2.34 (-0.09, 4.76)	2.45* (0.04, 4.86)
Physical activity (Non-leisure)						
	No		(ref)	(ref)	(ref)	(ref)
	Yes		-3.24** (-5.52, -0.95)	-3.27** (-5.55, -0.98)	-3.40** (-5.69, -1.12)	-3.36** (-5.63, -1.09)
Sitting duration (Quartile)						
	Q1		(ref)	(ref)	(ref)	(ref)
	Q2		0.68 (-2.25, 3.62)	0.69 (-2.25, 3.62)	0.72 (-2.21, 3.64)	0.68 (-2.22, 3.59)
	Q3		0.41 (-2.04, 2.87)	0.37 (-2.09, 2.83)	0.29 (-2.17, 2.75)	0.43 (-2.03, 2.89)
	Q4		-3.19* (-5.90, -0.47)	-3.20* (-5.92, -0.48)	-3.29* (-6.02, -0.55)	-2.80* (-5.53, -0.06)
Regular dental visits						
	No			(ref)	(ref)	(ref)
	Yes			0.50 (-1.57, 2.56)	0.44 (-1.62, 2.50)	0.31 (-1.73, 2.36)
Attend health screening						
	No			(ref)	(ref)	(ref)
	Yes			1.5 (-0.50, 3.49)	1.52 (-0.48, 3.51)	1.47 (-0.51, 3.45)
Age						
	< 75 years old				(ref)	(ref)
	≥ 75 years old				-1.89 (-4.30, 0.52)	-1.69 (-4.09, 0.71)
Gender						
	Male				(ref)	(ref)
	Female				1.48 (-1.18, 4.14)	1.46 (-1.18, 4.11)
Ethnicity						
	Chinese				(ref)	(ref)
	Malay/Indian/Others				3.98** (1.35, 6.60)	4.44*** (1.83, 7.06)
Marital status						
	Married				(ref)	(ref)
	Single/Separated/Divorced/Widowed				-0.39 (-2.75, 1.97)	-0.29 (-2.63, 2.06)
Multimorbidity						
	No condition					(ref)
	1 condition					1.57 (-1.23, 4.37)
	≥ 2 conditions					-2.76* (-5.27, -0.25)
Constant		84.11*** (79.54, 88.69)	85.14*** (80.21, 90.07)	84.49*** (79.46, 89.52)	82.81*** (77.48, 88.13)	83.06*** (77.47, 88.65)
R^2^		2%	4%	4%	6%	7%
Ratio of explained R^2^		29%	57%	57%	86%	100%
Change in ratio of explained R^2^		29%	29%	0%	29%	14%

*Notes*: CI = confidence interval.

*p < 0.05;

**p < 0.01;

***p < 0.001

## Discussion

Three measures of health were explored in this study: cognitive impairment, frailty, and self-rated health. We observed differences in association and overall variation explained by the five health determinants in all three health outcomes. Despite these differences, social determinants were found to contribute most of the overall variation found in the full model, suggesting the greater importance of social factors in influencing health. The most variation explained by social determinants was found in the model for cognitive impairment, followed by frailty, and lastly self-rated health.

Individuals with more privileged social circumstances were more likely to experience better health, consistent with other studies [33, 34]. There are several conceptual models supporting the relationship between social determinants and health outcomes [3, 35]. The WHO conceptual framework for action on the social determinants of health demonstrates how social determinants like income and education can influence an individual socioeconomic status which can, in turn, affects one’s health [3]. For instance, having a higher education level could open opportunities to job prospects with higher income, and this allows for easier access to healthcare services, improving health in the long term. The National Institute for Health and Clinical Excellence (NICE) conceptual framework for public health also recognises the significance of social determinants on health differentials at the individual and population levels [35].

Of the measures of social determinants, the level of educational attainment was most strongly associated with cognitive functioning and frailty. It was also the only variable that remained significant after adjusting for other health determinants. The association between education on frailty and cognitive impairment was also found in other studies, where individuals with higher education were associated with better health outcomes [36, 37]. However, household income was not a strong risk factor for poor health outcomes in this study, contrary to other studies [14, 38, 39]. A reason for this could be that most participants have already retired, and household income reflects only the household financial capability but not that of the individual [40, 41]. It does not include the financial resources accumulated throughout the years, and it is also likely that not all household members have equal access to the household income [40]. Thus, household income may be a weak representation of an individual’s social circumstances. In addition, 37% of participants chose not to disclose their household income, which was imputed with <S$2,000, the mean household income for all housing types.

Individuals who engaged in positive lifestyle behaviours were generally found to be associated with better health. This was also observed in prior studies [42, 43]. Physical activity was the lifestyle factor that was most strongly associated with cognitive impairment and frailty, similar to what was found in other studies [44, 45]. Interestingly, leisure physical activity was found to be protective against frailty, while non-leisure physical activity was found to be associated with greater odds of frailty. Frailty is multi-dimensional and comprises social, physical, and cognitive frailty. Physical frailty, including low gait speed and muscle strength, predicts social frailty, which further accelerates frailty and disability trajectory [46]. Studies analysing leisure and non-leisure physical activity also reported similar findings among older adults on depression and diabetes [47, 48]. Numerous studies suggest that individuals with lower socioeconomic positions were more likely to engage in unhealthy lifestyle practices, increasing the risks of morbidity [49, 50].

Health seeking behaviour contributed little to the overall variation in the adjusted models. Health seeking behaviour includes regular dental appointments and uptake of recommended health screening programmes such as blood stool tests, colonoscopy, and mammograms. Routine dental visits can help to prevent oral diseases and avoid developing problems related to activities of daily living such as eating, thus improving overall wellbeing [51]. Additionally, recommended health screenings enable the detection of preventable diseases which can be treated early, resulting in better health outcomes [30]. However, both regular dental visits and uptake of health screening programmes were not found to be significantly associated with any of the health outcomes. Due to the cross-sectional design of the study, we were unable to differentiate between individuals who attended the health screening programmes and dental visits voluntarily from individuals who were advised by a doctor because they were unwell or had a family history of the disease. As such, these two measures may not fully capture individuals’ health seeking behaviour.

Multimorbidity is described as the coexistence of two or more chronic conditions. Multiple chronic conditions accelerate the decline of functional and cognitive ability among older adults [52, 53]. Prior research suggested that a bidirectional association may exist between multimorbidity and frailty [54]. While an individual with multimorbidity may be at higher risk of frailty, frailty also increases the likelihood of a person developing multiple chronic conditions due to weakening resistance to internal and external stressors [54]. Additionally, individuals with multiple chronic conditions often have greater medical needs and may undergo more complex treatments, potentially affecting their physical and psychological state [55]. However, in our study, while multimorbidity was associated with increased odds of frailty and poorer self-rated health, it was not associated with cognitive impairment. This was in contrast with other studies that found an association between multimorbidity and poorer health outcomes [53, 56]. The lack of association may be due to inadequate power as only 15% of older adults had cognitive impairment in our study.

Several limitations should be noted. Most of the participants in our study had already stopped working as the retirement age in Singapore is 62. Wealth, instead of household income, is more reflective of their current economic status [40, 41]. It is an indication of the financial resources accumulated through time which may differ from current household income patterns [40]. However, as our study did not collect any data on wealth, we only included household income, in which 37% of them were either unaware of their household income or did not wish to disclose the amount. This was subsequently imputed with household income of <S$2,000 based on mean household income by housing type, which may have led to an underestimation in the higher income group in our study.

In addition, the cross-sectional design of this study does not allow for causal inference between determinants of health on older adults’ health. The findings of this study may be attenuated by survival bias as only older participants were included. Furthermore, all measures were self-reported. As such, the findings should be interpreted with caution.

Despite these limitations, the findings of this study highlight the importance of studying health determinants in understanding older adults’ health. Most intervention studies on frailty and cognitive impairment have yet to include interventions on social determinants of health. Psychosocial adversity, physiological decline with ageing, and unhealthy lifestyle and behaviours can culminate in a cascade of vascular and neurodegenerative changes causing shortening of health span. With the increase in ageing population across the world, more research should be done to understand the dynamics of multiple health determinants on older adults which demands a multidimensional, multidisciplinary and multisectoral approach [19]. Our study highlights the need for a multi-pronged approach in the prevention of frailty, cognitive impairment, and associated disability.
